# Patient Adherence to Screening for Lung Cancer in the US: A Systematic Review and Meta-analysis

**DOI:** 10.1001/jamanetworkopen.2020.25102

**Published:** 2020-11-16

**Authors:** Maria A. Lopez-Olivo, Kristin G. Maki, Noah J. Choi, Richard M. Hoffman, Ya-Chen Tina Shih, Lisa M. Lowenstein, Rachel S. Hicklen, Robert J. Volk

**Affiliations:** 1 Department of Health Services Research, The University of Texas MD Anderson Cancer Center, Houston; 2 Department of Internal Medicine, The Roy J. and Lucille A. Carver College of Medicine at the University of Iowa, Iowa City; 3 Research Medical Library, The University of Texas MD Anderson Cancer Center, Houston

## Abstract

**Importance:**

To be effective in reducing deaths from lung cancer among high-risk current and former smokers, screening with low-dose computed tomography must be performed periodically.

**Objective:**

To examine lung cancer screening (LCS) adherence rates reported in the US, patient characteristics associated with adherence, and diagnostic testing rates after screening.

**Data Sources:**

Five electronic databases (MEDLINE, Embase, Scopus, CINAHL, and Web of Science) were searched for articles published in the English language from January 1, 2011, through February 28, 2020.

**Study Selection:**

Two reviewers independently selected prospective and retrospective cohort studies from 95 potentially relevant studies reporting patient LCS adherence.

**Data Extraction and Synthesis:**

Quality appraisal and data extraction were performed independently by 2 reviewers using the Newcastle-Ottawa Scale for quality assessment. A random-effects model meta-analysis was conducted when at least 2 studies reported on the same outcome. Reporting followed the Preferred Reporting Items for Systematic Review and Meta-analyses (PRISMA) guideline.

**Main Outcomes and Measures:**

The primary outcome was LCS adherence after a baseline screening. Secondary measures were the patient characteristics associated with adherence and the rate of diagnostic testing after screening.

**Results:**

Fifteen studies with a total of 16 863 individuals were included in this systematic review and meta-analysis. The pooled LCS adherence rate across all follow-up periods (range, 12-36 months) was 55% (95% CI, 44%-66%). Regarding patient characteristics associated with adherence rates, current smokers were less likely to adhere to LCS than former smokers (odds ratio [OR], 0.70; 95% CI, 0.62-0.80); White patients were more likely to adhere to LCS than patients of races other than White (OR, 2.0; 95% CI, 1.6-2.6); people 65 to 73 years of age were more likely to adhere to LCS than people 50 to 64 years of age (OR, 1.4; 95% CI, 1.0-1.9); and completion of 4 or more years of college was also associated with increased adherence compared with people not completing college (OR, 1.5; 95% CI, 1.1-2.1). Evidence was insufficient to evaluate diagnostic testing rates after abnormal screening scan results. The main source of variation was attributable to the eligibility criteria for screening used across studies.

**Conclusions and Relevance:**

In this study, the pooled LCS adherence rate after a baseline screening was far lower than those observed in large randomized clinical trials of screening. Interventions to promote adherence to screening should prioritize current smokers and smokers from minority populations.

## Key Points

**Question:**

What is the of rate of adherence to lung cancer screening among high-risk individuals outside randomized clinical trials, and how does adherence differ across patient subgroups?

**Findings:**

In this systematic review and meta-analysis of 15 cohort studies with a total of 16 863 individuals, the pooled lung cancer screening adherence rate was 55%. Current smokers, patients of races other than White, those younger than 65 years, and those with less than a college education had lower adherence to screening.

**Meaning:**

These findings suggest that adherence to lung cancer screening is much lower than reported in large randomized clinical trials and is lower for current smokers and smokers from minority populations.

## Teaser

This systematic review and meta-analysis examines US lung cancer screening adherence rates, patient characteristics associated with adherence, and diagnostic testing rates after screening.

## Introduction

Screening high-risk current and former smokers for lung cancer with low-dose computed tomography (LDCT) reduces deaths from lung cancer.^1,2,3^ The US Preventive Services Task Force recommends annual screening with LDCT for individuals with a smoking history of at least 30 pack-years who currently smoke or have quit within the past 15 years, are between 55 and 80 years of age, and meet other eligibility criteria.^4^ Screening should continue annually until the person is no longer eligible.^5^

In the National Lung Screening Trial (NLST) and the Dutch-Belgian lung cancer screening (LCS) trial (the Nederlands-Leuvens Longkanker Screenings Onderzoek [NELSON] trial), adherence to subsequent screening was high. The NELSON trial’s adherence rates exceeded 90% during 4 screenings (final screening scan occurred 5.5 years after enrollment),^3^ and the NLST reported adherence rates greater than 95% during 3 annual screenings.^2^ Monitoring adherence rates for LCS outside clinical trials is important in understanding how LCS is being implemented in the US. This systematic review and meta-analysis examines LCS adherences rates outside the context of randomized clinical trials, differences in adherence rates among subgroups of patients, and diagnostic testing rates after screening.

## Methods

### Protocol and Registration

The protocol for this systematic review and meta-analysis is registered with PROSPERO. We followed the standards of the *Cochrane Handbook for Systematic Reviews of Interventions*^6^ and report our results according to the Preferred Reporting Items for Systematic Reviews and Meta-analyses (PRISMA) reporting guideline.^7^

### Eligibility Criteria

We included studies that reported LCS adherence rates in the US and/or determinants of LCS adherence. We considered prospective or retrospective studies that screened adult patients at any risk level of developing cancer who opted to initiate LCS and continued to undergo additional screening after the first LDCT. Because in some instances screening was not performed annually, from here on we use the term *periodic* to indicate a subsequent screening. We also considered any length of follow-up and setting. We excluded randomized clinical trials, studies without enough information to perform meta-analysis (ie, did not provide a denominator for adherence rates or determinants of adherence without the magnitude of association), and studies that reported on imaging techniques other than LDCT. For studies that reported the results in different years of the same cohort, we included the most updated report.

### Information Sources and Search Strategies

An experienced librarian (R.S.H.) searched 5 electronic databases: MEDLINE (via Ovid), Embase (via Ovid), Scopus, CINAHL, and Web of Science. eTable 1 in the Supplement gives the search strategy used for MEDLINE. Searches were limited to English-language articles published from January 1, 2011, through August 31, 2019. Our searches were updated via Ovid monthly autoalerts. We received new citations released by the databases up until February 29, 2020. The date restriction was imposed to ensure that only studies published after the NLST^2^ results were captured. The new citations were added for review before the analysis.

### Study Selection and Data Collection

Two members of the research team independently screened citations (K.G.M. and N.J.C.). Titles and abstracts were first screened to eliminate any citations not relevant to the study, and then the full text of the relevant citations were further screened for eligibility. Disagreements between reviewers were resolved by consensus or by a third person (M.A.L.-O.). Two members of the study team independently extracted data from the studies (K.G.M and N.J.C.), and any discrepancies were resolved by discussion. The data were also cross-checked for any errors by another author (M.A.L.-O.).

### Data Items

When available, we captured the following: (1) general study information, such as title, authors, follow-up, year, funding agency, study design, setting (ie, academic or community), definition of adherence, geography (ie, rural or urban), hospital type (ie, safety net or federally qualified health center), screening type (ie, integrated health center or need to refer patients for diagnostic testing), use of electronic health record, and number of patients analyzed; (2) characteristics of participants, such as age, sex, eligibility criteria, socioeconomic status, smoking status, and race/ethnicity; and (3) outcome variables, such as adherence rates of LCS, characteristics associated with adherence, and completion rates of recommended diagnostic testing after screening. Inclusion of data items was determined by possible associations between these factors and periodic LCS adherence. For instance, some federally qualified health centers serve individuals regardless of insurance status or ability to pay^8,9^; these factors may be associated with subsequent screening behavior.

### Risk of Bias in Individual Studies

Two authors (K.G.M. and N.J.C.) independently appraised the included studies for potential bias. Disagreements were resolved by consensus or by a third person (M.A.L.-O. or R.J.V.). We used the Newcastle-Ottawa Scale to assess the quality of nonrandomized studies in meta-analyses.^10^ The scale evaluates 3 domains of bias: selection, comparability, and measurement of outcomes. Each domain includes items that are scored with a star system.^10^ The maximum scores were 4 stars for the selection domain, 2 for the comparability domain, and 3 for the outcome (or exposure for case-control studies) domain. A total maximum score of 9 can be achieved, and a higher score indicates a lower risk of bias.

### Summary Measures

We analyzed data as reported in the studies. We determined adherence rates using the number of patients undergoing screening in each trial per time point as numerators. For the denominator, we considered all patients followed up for each time point (not everyone who receives a baseline scan is eligible for subsequent scans; for example, people may move to diagnostic testing or treatment or die). To quantify the association between adherence and variables of interest, we pooled the reported odds ratios (ORs) and 95% CIs. To determined diagnostic testing rates after screening, we used the number of patients undergoing any test or procedure with the purpose of diagnosis after an abnormal screening result as the numerator and all patients with abnormal results from LDCT as the denominator.

### Statistical Analysis

We used a random-effects model to calculate a combined estimate of LCS adherence rate and a 95% CI. For the pooled adherence rate, we used the Freeman-Tukey double arcsine transformation to stabilize variances and conducted a meta-analysis using inverse variance weights. Resulting estimates and 95% CI boundaries were back transformed into proportions. We used the generic inverse-variance method with a random-effects model when estimates of log ORs and SEs had been obtained from the included studies. When needed, we applied 1 divided by the OR for consistency of the referent group to pool estimates. For studies in which the number of events was provided, we calculated ORs and then converted them into log ORs and SEs. No attempts were made to contact authors of studies with missing data. When data were unclear or not provided for a given outcome, the study was not included in the analysis for the outcome, assuming that the data were missing at random.^11^ Heterogeneity of the data was formally tested by using the χ^2^ test, with *P* < .10 indicating significant heterogeneity; the *I*^2^ statistic results were also assessed (a value >50% may indicate substantial heterogeneity) and forest plots reviewed. All analyses were 2-sided and performed using Stata statistical software version 15 (StataCorp) and RevMan version 5.3 (The Cochrane Collaboration).

We used subgroup analysis to explore the length of follow-up and eligibility criteria as potential factors associated with heterogeneity. A metaregression was performed to evaluate the association between enrollment year and adherence rates. We planned to perform a funnel plot and a regression asymmetry test to assess small-study bias for the meta-analysis to identify the patient characteristics associated with adherence. Because of the small number of studies, a funnel plot and a regression asymmetry test to assess small-study bias for the meta-analysis could not be performed.

## Results

### Study Selection and Characteristics

**Figure 1.**
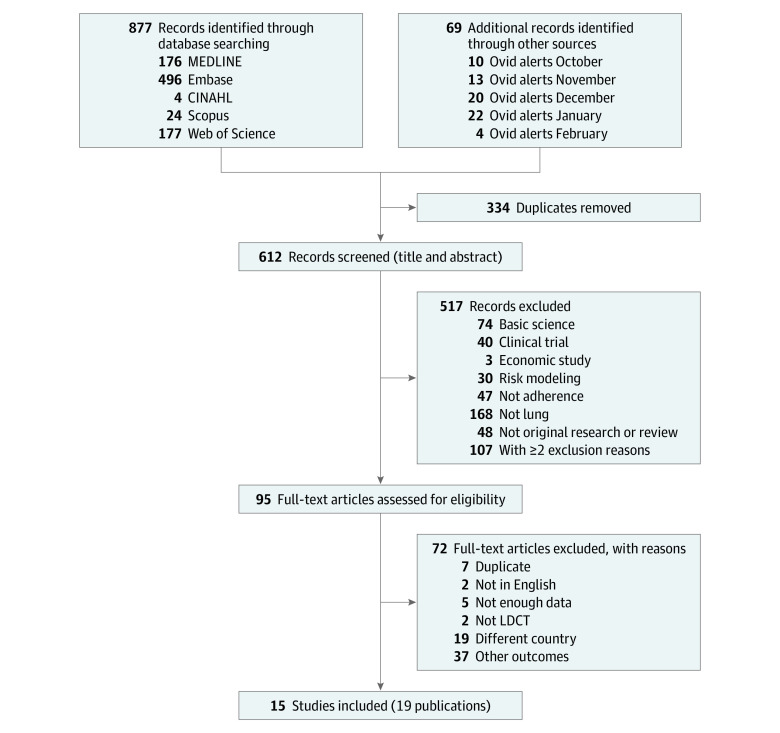
Flow Diagram of Study Disposition LDCT indicates low-dose computed tomography.

The flow diagram of study disposition is shown in Figure 1. Fifteen studies (19 publications) involving a total of 16 863 individuals were included in this systematic review.^12,13,14,15,16,17,18,19,20,21,22,23,24,25,26,27,28,29,30^

Ten studies were retrospective^12,13,14,15,17,19,22,23,24,25,26,27^ and 5 were prospective cohorts^16,18,20,21,28,29,30^ (Table 1). Eight studies^12,17,18,19,23,25,27,28^ were conducted in an academic setting and 7^13,14,15,16,20,22,29^ in a community setting. Aside from 1 study,^18^ adherence was evaluated for only the first subsequent screening. The length of follow-up ranged from 12 to 18 months, with 1 study^18^ reporting data to 36 months. Only 3 studies^14,27,29^ reported their funding sources.

**Table 1.** Characteristics of the Included Studies

Source	Participants, No.	Study type	Setting	Follow-up, mo	Definition of adherence	Recruitment period	Funding source
Alshora et al,^12^ 2018	901	Retrospective cohort	Academic	15	Completion of second screening within 3 mo of due date	Jan 12, 2012-Jun 12, 2013	NR
Bhandari et al,^13^ 2019	4500	Retrospective cohort	Community	12	NR	2016-2017	NR
Brasher et al,^14^ 2018	2106	Retrospective cohort	Community	15	Completion of second screening within 3 mo of due date	Jul 1, 2013-Jun 30, 2015	Exact Sciences, Oncimmune, Oncocyte, Olympus Medical
Cattaneo et al,^15^ 2018	1241	Retrospective cohort	Community	15	Completion of second screening within 3 mo of due date	Jan 2012-Oct 2015	NR
Gupta et al,^16^ 2014	356	Prospective cohort	Community	12	Completion of additional screening within any time frame	Jun 1, 2011-May 30, 2013	NR
Hirsh et al,^17^ 2019	259	Retrospective cohort	Academic	18	Completion of second screening within 6 mo of due date	Jul 1, 2014-Dec 31, 2016	NR
Kaminetsky et al,^18^ 2019	1181	Prospective cohort	Academic	12^a^	Completion of second, third, and fourth annual screening	Dec 2012-Dec 2016	NR
Plank et al,^19^ 2018	825	Retrospective cohort	Academic	15	Completion of second screening within 3 mo of due date	NR	NR
Porubcin et al,^20,21^ 2015, 2017	466	Prospective cohort	Community	NR	NR	Apr 2013-Jun 2016	NR
Sakoda et al,^22^ 2018	145	Retrospective cohort	Community	10-14	Completion of second screening within 10-14 mo of due date	Jul 2014-Jun 2015	NR
Spalluto et al,^23,24^ 2018, 2020	319	Retrospective cohort	Academic	15	Completion of second screening within 3 mo of due date	Jan 1, 2014-Sep 30, 2016	NR
Thayer et al,^25,26^ 2019	645	Retrospective cohort	Academic	15	Completion of second screening within 3 mo of due date	2012-Apr 30, 2017^b^	NR
Vachani et al,^27^ 2019	375	Retrospective cohort	Academic	11-30 mo	Completion of additional screening within any time frame	Jan 1, 2014-Dec 31, 2016	NCI
Wildstein et al,^28^ 2011	3387^c^	Prospective cohort	Academic	18	Completion of second screening within 6 mo of due date	Self-pay: 1999-2003; no pay: 2001-2002	NR
Young et al,^29,30^ 2015	157	Prospective cohort	Community	12	Completion of additional screening within any time frame	Started in 2010; end date NR	Camino Hospital Trust, Synergenz Bioscience Ltd

Abbreviations: NCI, National Cancer Institute; NR, not reported.

^a^ The study also reported data at 24 and 36 months from initial lung cancer screening.

^b^ Month and day of start date 2 not reported.

^c^ Results are presented for 2 cohorts: no pay (n = 1304) and self-pay (n = 2083).

The mean age of participants ranged from 50 to 75 years, the percentage of men ranged from 42% to 65%, the percentage of current smokers ranged from 42% to 76%, and the mean pack-year smoking history ranged from 32 to 53 pack-years (Table 2).^16,17,18,19,20,21,22,23,24,25,26,27,28,29,30^ Eligibility criteria varied across studies, with several reporting broad criteria not reflecting current guidelines.^13,23,24,28,29,30^ Two studies reported results for separate cohorts: Hirsh et al^17^ subdivided individuals into those who received a screening reminder and those who did not, and Wildstein et al^28^ applied eligibility criteria for screening to 2 cohorts that differed from US Preventive Services Task Force criteria or guidance from the Centers for Medicare & Medicaid Services. Specifically, in the self-pay cohort, individuals were 40 years or older and had a smoking history of at least 1 pack-year. For the non–self-pay cohort, individuals were at least 60 years of age and had a smoking history of at least 10 pack-years.

**Table 2.** Characteristics of the Participants in the Included Studies

Source	Age, y	Male sex, No. (%)	Race/ethnicity	Insurance	Current smokers, No. (%)	Pack-years, mean (SD)	Eligibility criteria
Alshora et al,^12^ 2018	Range, 50-74	503 (56)	>95% White	Not reported	414 (46)	Not reported	NCCN guidelines^a^
Bhandari et al,^13^ 2019	Median, 64	2070 (46)	Not reported	Not reported	3105 (69)	52	All lung cancer screening patients within a Kentucky health system
Brasher et al,^14^ 2018	Mean, 66^b^; range, 55-80	Not reported	Not reported	Conducted within VA	Not reported	Not reported	Ages 55-80 y, ≥30–pack-year smoking history, including former smokers who had quit within 15 y
Cattaneo et al,^15^ 2018	Ranges, <50 (n = 15), 55-77 (n = 1194), 78-80 (n = 25), >80 (n = 7)	590 (48)	White (n = 1084), African American (n = 126), other (n = 18), race not reported (n = 12)^c^	Private (n = 617), Medicare (n = 565), Medicaid (n = 17), not reported (n = 42)	609 (49)^d^	40^b^	NLST^e^
Gupta et al,^16^ 2014	Mean, 62; range, 53-71	150 (42)	White (n = 328), African American (n = 21)	Not reported	Not reported	Not reported	NLST^e^
Hirsh et al,^17^ 2019	Reminder: mean (SD), 64.1 (5.6)	Reminder: 116 (57)	Reminder: White (n = 172), no reminder: White (n = 42)	Reminder: government (n = 151), private (n = 49), other (n = 5)	Reminder: 113 (55)	Reminder: 48.5 (17.8)	CMS guidelines^f^
	No reminder: mean (SD), 64.3 (6.1)	No reminder: 32 (59)		No reminder: government (n = 40), private (n = 11), other (n = 3)	No reminder: 29 (54)	No reminder: 49.1 (17.3)	
Kaminetsky et al,^18^ 2019	Mean (SD), 64 (16.2)	569 (48)	White (n = 271), African American (n = 371), Hispanic (n = 365), Asian (n = 8), race not reported (n = 166)	Medicare (n = 658), Medicaid (n = 248)	843 (71)	45	NLST^e^
Plank et al,^19^ 2018	Mean, 60	495 (60)	Not reported	NA	347 (42)	46 (24)	NCCN guidelines^a^
Porubcin et al,^20,21^ 2015, 2017	Median, 64^b^; range, 55-80	234 (50)	Not reported	Not reported	Not reported	≥30	Ages 55-80 y, ≥30–pack-year smoking history, including former smokers who had quit within 15 y
Sakoda et al,^22^ 2018	Median, 66^b^	88 (61)	White (n = 103)	Conducted within Kaiser Permanente	110 (76)	Not reported	Had baseline screen from 2014-2015, continuous health plan enrollment for ≥14 mo after baseline
Spalluto et al,^23,24^ 2018, 2020	Ranges, <55 (n = 6), 55-59 (n = 71), 60-64 (n = 81), 65-69 (n = 102), 70-74 (n = 47), ≥75 (n = 12)	162 (51)	White (n = 277), African American (n = 23), Hispanic or Latino (n = 4), other or missing (n = 19)	Not reported	Not reported	Not reported	Baseline LDCT between 2014 and 2016, baseline Lung-RADS score of 1 or 2, 12-mo follow-up recommendation
Thayer et al,^25,26^ 2019	Mean, 63	419 (65)	Not reported	Not reported	342 (53)	53^b^	Had a baseline screen from 2012-2017
Vachani et al,^27^ 2019	Ranges, 55-60 (n = 107), 61-65 (n = 113), 66-70 (n = 106), 71-75 (n = 49)	206 (55)	White (n = 205), African American (n = 143), Hispanic (n = 2), Asian (n = 6), multiple (n = 8), race not reported (n = 11)	Not reported	Not reported	Not reported	Baseline LDCT 2014-2016, ages 55-75 y at baseline, Lung-RADS score of 1 or 2 at baseline, at least 1 primary care visit at Penn Medicine before and after baseline
Wildstein et al,^28^ 2011	Self-pay: mean, 59; range, 40-87	Self-pay: 1005 (48)	Self-pay: White (n = 1983), African American (n = 43), Hispanic (n = 20), Asian (n = 20), other (n = 17)	Not reported	Self-pay: former, 1364 (65)	Self-pay: 32^b^	Self-pay: ≥40 y of age, ≥1–pack-year smoking history, no prior cancer, no CT in prior 3 y
	No pay: mean, 66; range, 60-92	No pay: 598 (46)	No pay: White (n = 1058), African American (n = 148), Hispanic (n = 67), Asian (n = 29), other (n = 2)	Not reported	No pay: former, 875 (67)	No pay: 40^b^	No pay: age ≥60 y, ≥10–pack-year smoking history, no prior cancer (other than nonmelanotic skin cancer), no CT in prior 3 y
Young et al,^29,30^ 2015	Range, >50	Not reported	Not reported	Not reported	Not reported	Not reported	>50 y Of age, ≥20–pack-year history, volunteered for CT screening (using the International Early Lung Cancer Action Program)

Abbreviations: CMS, Centers for Medicare & Medicaid Services; CT, computed tomography; LDCT, low-dose computed tomography; Lung-RADS, categorization tool designed to standardize the reporting of screening-detected lung nodules; NA, not applicable; NCCN, National Comprehensive Cancer Network; NLST, National Lung Screening Trial; VA, Veterans Affairs.

^a^ Individuals 50 years or older with a 20 or more pack-year history of smoking tobacco and other risk factors.

^b^ Values are medians.

^c^ Numbers reported in the original article, in which values did not sum to the total sample size of 1241.

^d^ Former: n = 598; not reported: n = 34.

^e^ Current or former heavy smokers 55 to 74 years of age. Participants were required to have a smoking history of at least 30 pack-years and were current or former smokers without signs, symptoms, or history of lung cancer.

^f^ Age of 55 to 74 years; asymptomatic (no signs or symptoms of lung disease); tobacco smoking history of at least 30 pack-years (1 pack-year equals smoking 1 pack per day for 1 year; 1 pack equals 20 cigarettes); current smoker or one who has quit smoking within the past 15 years; and a lung cancer screening counseling and shared decision-making visit.

### Risk of Bias Within Studies

Ten studies^12,14,15,16,17,18,19,20,23,29^ (67%) reported an adequate selection of the cohort, and 12 studies^12,13,14,15,16,17,19,20,23,27,28,29^ (80%) were judged to have adequately ascertained that participants underwent screening. Ten studies^12,14,15,16,17,23,25,27,28,29^ (67%) were judged to have a low risk of confounder bias. Thirteen studies^12,13,14,15,16,17,19,20,22,23,27,28,29^ (87%) confirmed screening adherence through medical records or large database records. However, 12 studies^12,14,15,16,17,18,20,22,23,25,27,28^ (80%) did not have a follow-up time that was long enough to adequately assess periodic adherence beyond 1 year. All of the studies reported loss-to-follow-up rates greater than 20% (eTable 2 in the Supplement).

### Adherence Rates

**Figure 2.**
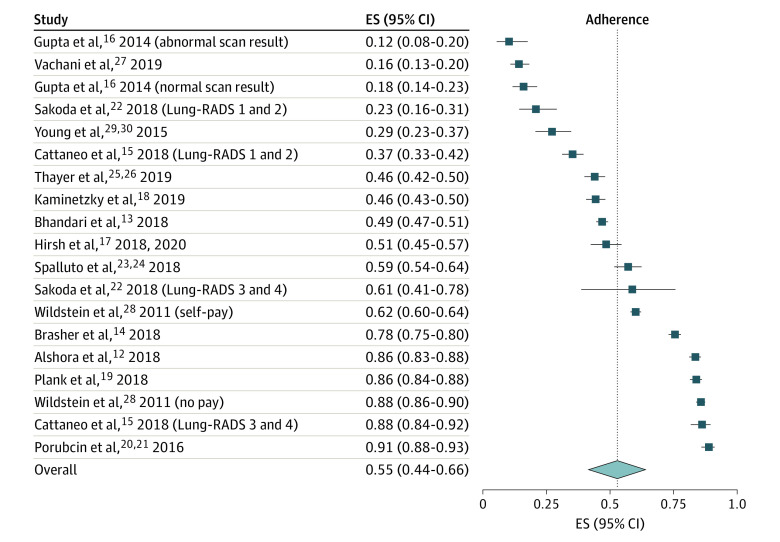
Lung Cancer Screening Adherence Rates at Any Time Point Lung-RADS is a categorization tool designed to standardize the reporting of screening-detected lung nodules. This figure shows the adherence rates reported per study. The first column represents the studies included in the analysis. The adherence rates were sorted from lowest to highest. The boxes represent the adherence rate reported per study after initial lung cancer screening (second screening regardless of the time point used). The horizontal lines represent 95% CIs. The diamond represents the overall adherence rate (pooled adherence rate) and the width of the diamond the 95% CI. The dotted line indicates where the overall effect estimate (pooled adherence rate) lies. ES indicates effect size.

The pooled LCS adherence rate across all follow-up periods was 55% (95% CI, 44%-66%) (Figure 2). Screening adherence rates across studies ranged from 12% (95% CI, 8%-20%) to 91% (95% CI, 88%-93%). eFigure 1 in the Supplement shows the adherence rates by follow-up times. Four studies^13,16,18,29^ reported screening adherence 12 months after baseline scan; the pooled rate for those studies was 30% (95% CI, 18%-44%). Six studies^12,14,15,19,23,25^ reported adherence 15 months after baseline scan; the pooled rate was 70% (95% CI, 55%-84%). Two studies^17,28^ reported adherence 18 months after baseline scan; the pooled rate was 68% (95% CI, 45%-88%). Reports of adherence at 24 and 36 months were provided by 1 study^18^ (38% at 24 months and 28% at 36 months were eligible for subsequent screening based on completing the previous year’s scan). eFigure 2 in the Supplement shows the results of studies that reported adherence rates within a period of 10 to 14 months^22^ and 11 to 30 months^27^ from baseline scan. One of these studies^27^ also reported adherence rates at any time point for those people with at least 1 additional screening and people with at least 2 additional screenings.

### Patient Characteristics Associated With Adherence Rates

Table 3 gives the patient characteristics associated with adherence rates. Smoking status was associated with adherence rates, and patients categorized as current smokers were less likely to adhere to LCS compared with former smokers (OR, 0.70; 95% CI, 0.62-0.80). White race was associated with higher adherence rates compared with races other than White (OR, 2.0; 95% CI, 1.6-2.6). Age was evaluated in 4 studies,^12,22,23,28^ and people 65 to 73 years of age were more likely to adhere than people 50 to 64 years of age (OR, 1.4; 95% CI, 1.0-1.9).^12,23^ Education was evaluated in 2 cohorts (1 study^28^), and completion of 4 years or more of college was associated with increased adherence compared with not completing college (OR, 1.5; 95% CI, 1.1-2.1). No other patient characteristics that were reported by 2 or more studies were statistically significantly associated with LCS adherence.

**Table 3.** Patient Characteristics Associated With Adherence Rates

Characteristic	Studies, No.	Odds ratio (95% CI)
Sex (female vs male)	4 studies (5 estimates)^12,15,22,28^	1.0 (0.8-1.3)
Smoking status (current vs former)	4 studies (5 estimates)^12,15,25,28^	0.7 (0.6-0.8)
Race/ethnicity (White vs other than White)	4 studies (5 estimates)^15,22,23,28^	2.0 (1.6-2.6)
Age, y		
60-69 (vs ages 40-59)	2 studies^23,28^	2.2 (0.6-7.9)
65-73 (vs ages 50-64)	2 studies^12,23^	1.4 (1.0-1.9)
>70 (vs ages 40-59)	2 studies^23,28^	1.7 (0.8-3.5)
>70 (vs ages 60-69)	2 studies^23,28^	0.7 (0.5-0.9)
Older (vs median age)	1 studies^25^	1.5 (1.0-2.3)
Insurance		
Private vs Medicare	1 study^15^	0.9 (0.6-1.3)
Private vs Medicaid	1 study^15^	2.5 (0.5-11.8)
Reminders		
Reminder (any) vs no reminder	1 study^17^	192.4 (11.7-3160.9)
Reminder from PCP vs no reminder	1 study^17^	327.0 (18.8-5693.3)
Reminder from nurse navigator vs no reminder	1 study^17^	164.8 (10.0-2717.7)
Educational level (≥4 y of college vs did not complete college)	1 study (2 estimates)^28^	1.5 (1.1-2.1)
Family history of lung cancer (vs no history)	1 study^28^	1.0 (0.8-1.3)
Findings		
Findings at baseline (semipositive or positive vs negative)	3 studies (4 estimates)^12,22,28^	1.6 (0.7-3.5)
Baseline results (probably benign vs suspicious)	1 study^12^	2.6 (0.6-11.2)
Risk		
Patient-perceived risk of developing cancer (high vs not high)	1 study (2 estimates)^28^	6.1 (0.04-1005.3)
Risk: gene-based risk algorithm, combining clinical risk variables with risk SNP genotypes to derive a composite lung cancer risk score (very high risk vs high to moderate risk)	1 study^29,30^	2.1 (0.9-4.7)

Abbreviations: PCP, primary care physician; SNP, single-nucleotide polymorphism.

### Additional Analyses

Subgroup analysis was conducted to explore differences on the adherence rates per eligibility criteria used (eFigure 3 in the Supplement). We observed a difference only in a study^28^ that included patients older than 80 years. After eliminating studies in which ORs had to be calculated from the number of events, the direction and the magnitude of the estimates for smoking status (OR, 0.69; 95% CI, 0.58-0.81) and ethnicity (OR, 2.0; 95% CI, 1.4-3.0) remained the same. In addition, the pooled adherence rate was not influenced by the enrollment year. Evidence was insufficient to evaluate diagnostic testing rates after abnormal screening scan results.

## Discussion

This systematic review and meta-analysis examined high-risk patients’ adherence to periodic LCS reported in cohort studies. It provides an indication of how successfully LCS is being implemented in the US since the release of the NLST’s main findings and subsequent recommendations endorsing screening with LDCT. We found that periodic screening rates for lung cancer were much lower—55% in our overall pooled analysis—than the rates reported in clinical trials. In addition, the rates varied widely, from 12% to 91%, and were higher when longer periods between initial and subsequent screenings were used.

Given the overall low rates of cancer screening adherence within the US population^31,32,33,34^ and among high-risk individuals,^35,36^ it is not surprising that LCS adherence was lower than that seen within the controlled setting of clinical trials.^37^ Results from the 2018 Behavioral Risk Factor Surveillance System survey indicate that approximately 68.8% of eligible adults in the US are up to date on colon cancer screening, an increase from previous years.^38^ According to data from the 2018 National Health Interview Survey, approximately 70% of the eligible population of women underwent breast cancer screening within the past 2 years and approximately 80% of eligible women received cervical cancer screening; this finding sharply contrasts with the 5.9% of eligible adults who underwent LCS in 2015.^39^ However, these estimates reflect only whether an individual has undergone screening within a window recommended by screening guidelines and are not indicators of long-term adherence.

The higher screening uptake and adherence rates for colon and breast cancer compared with lung cancer are the results of these tests being available and recommended for many years, and a great deal of effort has gone into educating patients,^40^ working with practitioners,^41^ and understanding factors that relate to screening behaviors.^42,43,44^ In contrast, LDCT for LCS is a relatively nascent field^45^ with most intervention efforts still focusing on increasing uptake and acceptability among patients and practitioners^46,47^ rather than promoting the importance of annual adherence.

Important differences between patient subgroups were found in this review. Current smokers were less likely to adhere to LCS than former smokers. This finding aligns with previous research reporting lower rates of cancer screening among eligible current smokers (compared with never smokers).^48,49^ Stigma may be a key barrier for LCS, with patients feeling judged and blamed and therefore delaying early screening.^50^ Prior work^51^ suggests that lung cancer stigma is a multilayered issue that spans individual and societal levels and includes placing blame on the individual for smoking as well as public attitudes and policies. Furthermore, patients have reported feeling as though some health care professionals do not understand how their smoking was affected by the culture and period in which they have lived.^50^

White people were more likely to adhere to periodic LCS than people of other races, a finding consistent with disparities seen by others^49^ and for other cancer screenings and diagnostic testing.^52,53^ Reasons for this disparity are unclear and may relate to insurance status and access to screening facilities, among other factors. Previous research has also found racial/ethnic disparities in screening, including for breast cancer,^54,55^ colorectal cancer,^56,57^ and follow-up diagnostic testing after a positive prostate cancer screening test result.^58^ Similarly, prior work^52^ has found a longer screening interval between prostate-specific antigen testing and prostate cancer diagnosis in Black men compared with White men.

This review has implications for future research and updates to current screening recommendations. Extending the recommended interval between lung cancer screenings^59^ has the potential to increase screening adherence, reduce false-positive test results, and decrease screening costs. Future research should investigate the optimal screening interval that balances the harm-benefit tradeoffs of LCS. There is also interest in the role of risk-based screening in lung cancer.^60^ Because smoking status is an important risk factor for lung cancer, concerns about adherence will be even greater if screening recommendations prioritize identification of high-risk current smokers. Interventions should be directed toward increasing LCS adherence among several key groups: current smokers, patients of races other that White, and patients with lower levels of education. Finally, data are needed to determine the adherence with diagnostic testing among patients with abnormal scan results and adherence with curative treatment for those diagnosed with a stage I or II cancer.

### Limitations

This review has limitations. We only included studies that were conducted in the US. The follow-up period was shorter than seen in the clinical trials, with most studies^12,13,14,15,16,17,19,20,22,23,25,27,28,29^ reporting a single follow-up screening. Information about subsequent adherence beyond 1 additional screening was not available, with 1 report^18^ of adherence beyond 18 months. We could not rule out influences of selective reporting of positive or negative results. Finally, there was heterogeneity of the LCS eligibility criteria across the included studies, suggesting that future research should consider how differences in patients’ risk of lung cancer impacts their adherence to screening.

## Conclusions

In this study, rates of LCS adherence in the US published in the literature varied widely and were lower than seen in the controlled setting of clinical trials. Few studies reported adherence beyond 1 subsequent screening after baseline. Although there is concern that screening rates nationally are low,^61^ equally important is the need for interventions to improve adherence to screening for current smokers and smokers from minority populations to fully realize the benefits of early detection of lung cancer.

## Notes

Supplement. **eTable 1.** MEDLINE (Ovid) Search Strategy Run on August 29, 2019 **eTable 2.** Risk of Bias Within Studies Assessed With the Newcastle-Ottawa Scale **eFigure 1.** Lung Cancer Screening Adherence Rates by Follow-up Times (12, 15, 18, 24, and 36 Months) **eFigure 2.** Lung Cancer Screening Adherence Rates by Follow-Up Times (Unspecified and Those Provided as Ranges) **eFigure 3.** Lung Cancer Screening Adherence Rates by Study Eligibility Criteria
