# Supplementary material for: Patient Adherence to Screening for Lung Cancer in the US: A Systematic Review and Meta-analysis
Source: JAMA Netw Open. 2020 Nov 16;3(11):e2025102. doi: 10.1001/jamanetworkopen.2020.25102 (PMC7670313; doi:10.1001/jamanetworkopen.2020.25102)
Supplement: Supplement. — eTable 1. MEDLINE (Ovid) Search Strategy Run on August 29, 2019 eTable 2. Risk of Bias Within Studies Assessed With the Newcastle-Ottawa Scale eFigure 1. Lung Cancer Screening Adherence Rates by Follow-up Times (12, 15, 18, 24, and 36 Months) eFigure 2. Lung Cancer Screening Adherence Rates by Follow-Up Times (Unspecified and Those Provided as Ranges) eFigure 3. Lung Cancer Screening Adherence Rates by Study Eligibility Criteria [file jamanetwopen-e2025102-s001.pdf]

Supplementary Online Content

Lopez-Olivo MA, Maki KG, Choi NJ, et al. Patient adherence to screening for lung cancer in the US: a systematic review and meta-analysis. *JAMA Netw Open*. 2020;3(11):e2025102. doi:10.1001/jamanetworkopen.2020.25102

eTable 1. MEDLINE (Ovid) Search Strategy Run on August 29, 2019

eTable 2. Risk of Bias Within Studies Assessed With the Newcastle-Ottawa Scale

eFigure 1. Lung Cancer Screening Adherence Rates by Follow-up Times (12, 15, 18, 24, and 36 Months)

eFigure 2. Lung Cancer Screening Adherence Rates by Follow-Up Times (Unspecified and Those Provided as Ranges)

eFigure 3. Lung Cancer Screening Adherence Rates by Study Eligibility Criteria

This supplementary material has been provided by the authors to give readers additional information about their work.

© 2020 Lopez-Olivo MA et al. *JAMA Network Open.*

eTable 1. MEDLINE (Ovid) Search Strategy Run on August 29, 2019

Line	Terms used
1	exp LUNG NEOPLASMS/
2	Lung*.ti.
3	or/1-2
4	EARLY DETECTION OF CANCER/
5	MASS SCREENING/
6	or/4-5
7	3 and 6
8	exp LUNG NEOPLASMS/pc
9	exp TOMOGRAPHY, X-RAY COMPUTED/
10	("computed tomograph*" or LDCT or "low dose CT").ti,kf,ab.
11	8 and (9 or 10)
12	((screen* or (early adj3 detect*)) and lung* and (cancer* or neoplas* or adenocarcinoma*)).ti,kf,ab.
13	((screen* or (early adj3 detect*)) and (SCLC or NSCLC)).ti,kf,ab.
14	((screen* or (early adj3 detect*)) adj5 lung* adj5 (cancer* or neoplas* or adenocarcinoma* or SCLC or NSCLC)).ab. /freq=2
15	((screen* or (early adj3 detect*)) adj5 (SCLC or NSCLC)).ab. /freq=2
16	or/12-15
17	7 or 11 or 16 [Lung Cancer Screening]
18	exp Patient Compliance/
19	(adher* or complian* or nonadher* or non-adher* or comply* or complies or complied or complian* or noncomply* or noncomplie* or noncomplian* or non-comply* or non-complie* or non-complian* or overadher* or over-adher* or overcomply* or over-comply* or overcomplie* or over-complie* or overcomplian* or over-complian*).ab,ti.
20	((predictor* or determinant* or "associated factor*") adj8 (adher* or nonadher* or non-adher* or comply* or complies or complied or complian* or noncomply* or noncomplie* or noncomplian* or non-comply* or non-complie* or non-complian* or overadher* or over-adher* or overcomply* or over-comply* or overcomplie* or over-complie* or overcomplian* or over-complian*)).ab,ti.
21	or/18-20 [Compliance]
22	17 and 21
23	limit 22 to (english language and yr="2011 -Current")

© 2020 Lopez-Olivo MA et al. *JAMA Network Open.*

eTable 2. Risk of Bias Within Studies Assessed With the Newcastle-Ottawa Scale

Study	Risk of Bias Assessment								
	Selection				Comparability	Outcome			Overall score
	Exposed	Unexposed	Exposure	Outcome		Assessment	Follow-Up	Attrition	
Alshora 2018	✸		✸	✸	✸	✸			5
Bhandari 2018			✸	✸		✸	✸		5
Brasher 2018	✸		✸	✸	✸	✸			6
Cattaneo 2018	✸		✸	✸	✸ ✸	✸			5
Gupta 2014	✸		✸		✸	✸			3
Hirsh 2019	✸		✸		✸	✸			3
Kaminetsky 2019	✸								1
Plank 2018	✸		✸	✸		✸	✸		5
Porubcin 2016	✸		✸			✸			3
Sakoda 2018				✸		✸			2
Spalluto 2018	✸		✸	✸	✸	✸			4
Thayer 2019					✸				2
Vachani 2019			✸	✸	✸	✸			4
Wildstein 2011_self-pay			✸		✸	✸			3
Young 2015	✸		✸	✸	✸	✸	✸		6

© 2020 Lopez-Olivo MA et al. *JAMA Network Open.*

eFigure 1. Lung Cancer Screening Adherence Rates by Follow-up Times (12, 15, 18, 24, and 36 Months)

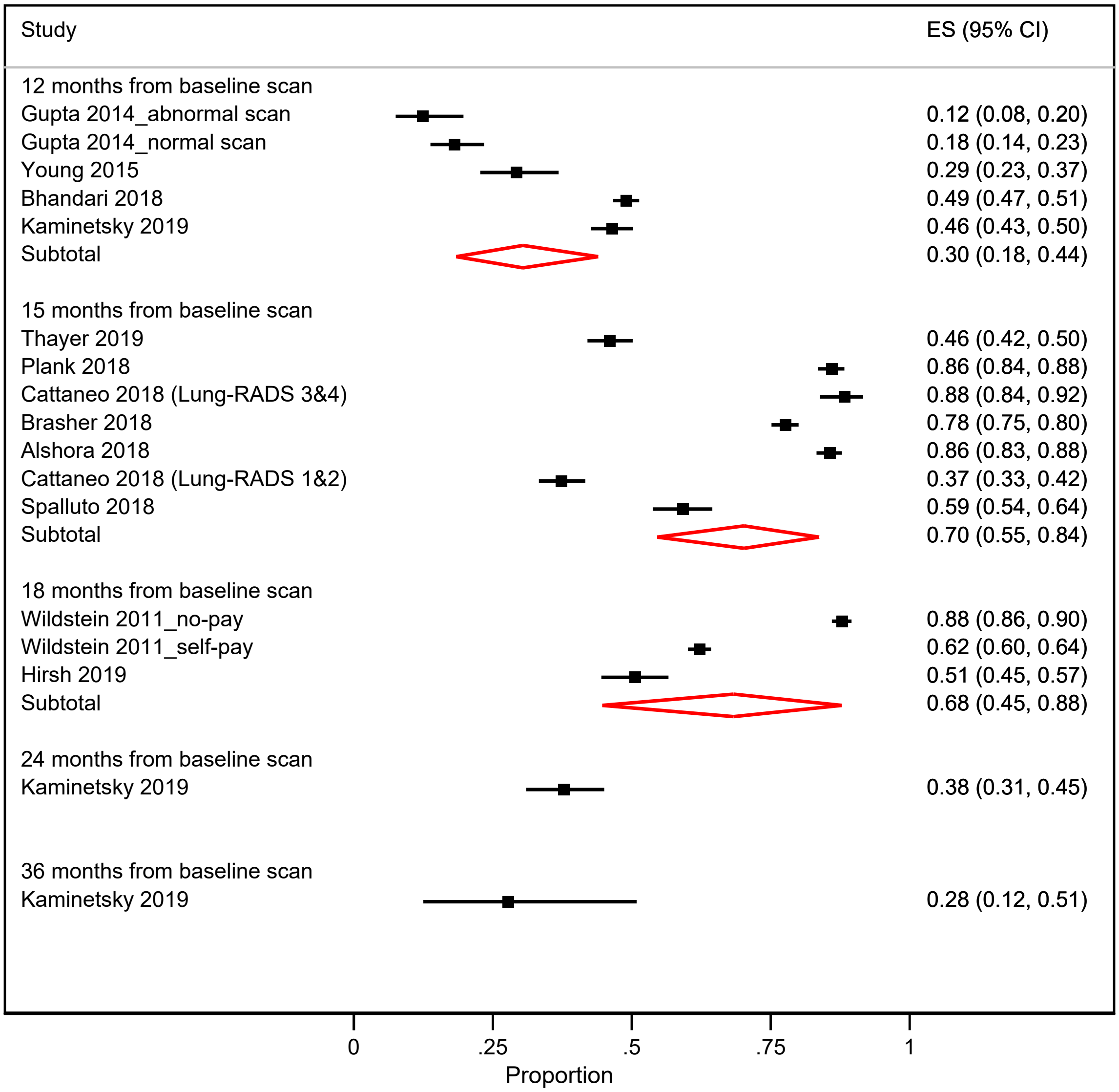

This figure shows the adherence rates reported per study according to the follow-up time. The first line in the study column indicates the subgroup. The adherence rates were sorted from lowest to highest. The black boxes represent the adherence rate reported per study after initial lung cancer screening (second screening regardless of the timepoint used). The black lines represent the 95% confidence interval. The red diamonds represent the pooled adherence rate for the subgroup and the width of the diamond respresent the 95% confidence interval.

© 2020 Lopez-Olivo MA et al. *JAMA Network Open.*

eFigure 2. Lung Cancer Screening Adherence Rates by Follow-Up Times (Unspecified and Those Provided as Ranges)

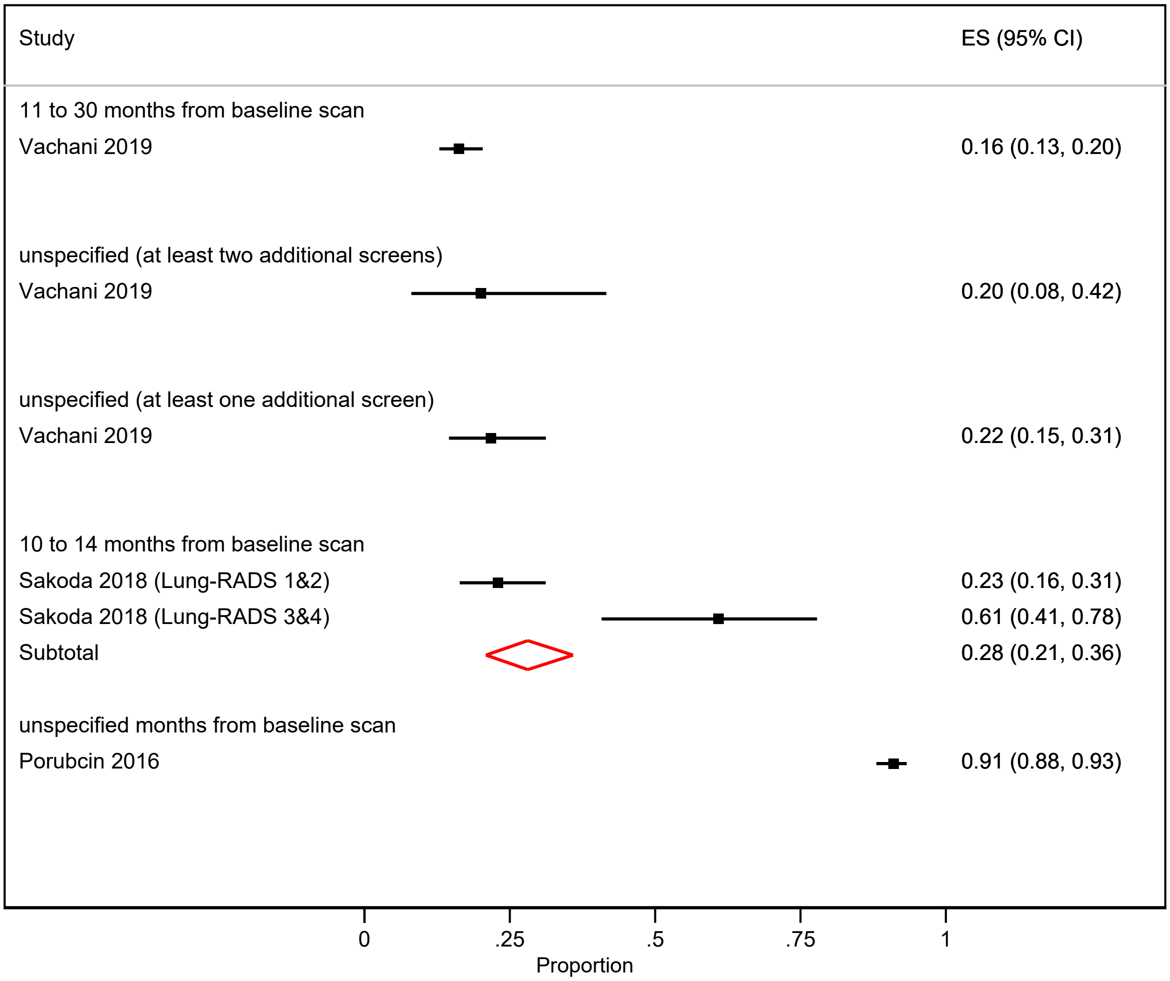

This figure shows the adherence rates reported per study for those studies not providing the follow-up time (when was the subsequent screening done) or reporting their follow-up time in rages (screening done in different times). The first line in the study column indicates the subgroup. The adherence rates were sorted from lowest to highest. The black boxes represent the adherence rate reported per study after initial lung cancer screening (second screening regardless of the timepoint used). The black lines represent the 95% confidence interval. The red diamonds represent the pooled adherence rate for the subgroup and the width of the diamond respresent the 95% confidence interval.

© 2020 Lopez-Olivo MA et al. *JAMA Network Open.*

e**Figure 3.** Lung Cancer Screening Adherence Rates by Study Eligibility Criteria

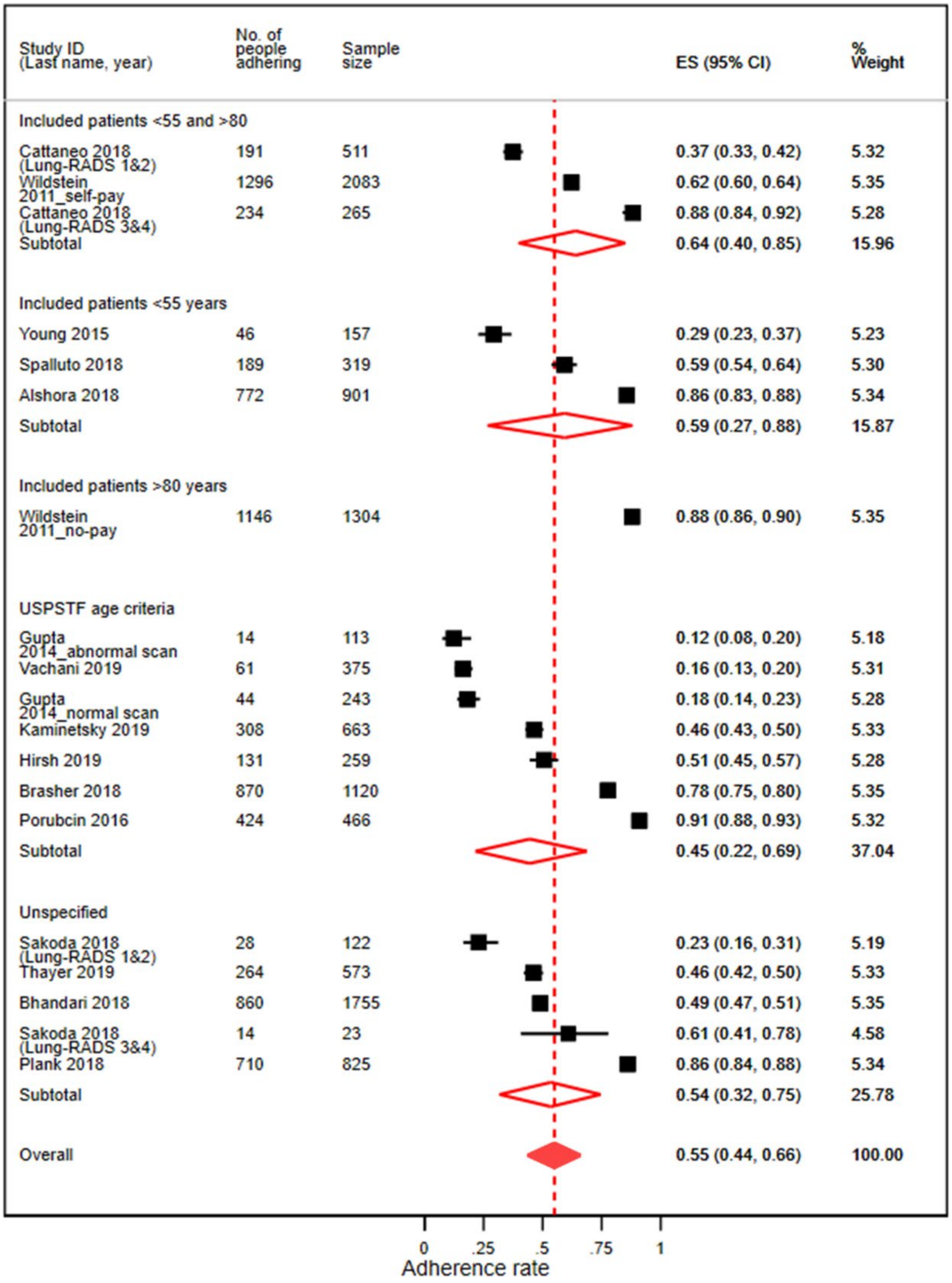

This figure shows the adherence rates reported per study their eligibility criteria. The first line in the study column indicates the subgroup (e.g., studies in which participants were between 55 and 80 years old, studies in which participants younger than 55 were included, etc.). The second and third columns indicate the counts (number of people adhering/number of sample size). The

© 2020 Lopez-Olivo MA et al. *JAMA Network Open.*

fourth column indicates the adherence rates, which were sorted from lowest to highest. The fifth column indicates the weight of the study (contribution to the overall adherence rate). The black boxes represent the adherence rates reported per study after initial lung cancer screening (second screening regardless of the time point used). The horizontal black lines represent the 95% confidence interval. The red diamonds represent the pooled adherence rate for the subgroup and the width of the diamond represent the 95% confidence interval. The last solid red diamond represent the overall (pooled) adherence rate combining all studies (independently of the subgroup). The dotted vertical line indicates the overall adherence rate.

© 2020 Lopez-Olivo MA et al. *JAMA Network Open.*
